# Ceanothanes Derivatives as Peripheric Anionic Site and Catalytic Active Site Inhibitors of Acetylcholinesterase: Insights for Future Drug Design

**DOI:** 10.3390/ijms25137303

**Published:** 2024-07-03

**Authors:** Sofía Pastene-Burgos, Evelyn Muñoz-Nuñez, Soledad Quiroz-Carreño, Edgar Pastene-Navarrete, Luis Espinoza Catalan, Luis Bustamante, Julio Alarcón-Enos

**Affiliations:** 1 Grupo de Investigación Química y Biotecnología de Productos Naturales Bioactivos, Laboratorio de Síntesis y Biotransformación de Productos Naturales, Departamento de Ciencias Básicas, Facultad de Ciencias, Universidad del Bío-Bío, Chillán 3800708, Chile; sofiapastene@gmail.com (S.P.-B.); evdmunoz@gmail.com (E.M.-N.); sole.m.quiroz.c@gmail.com (S.Q.-C.); edgar.pastene@gmail.com (E.P.-N.); 2 Departamento de Química, Universidad Federico Santa María, Valparaíso 2340000, Chile; luis.espinozac@usm.cl; 3 Departamento Análisis Instrumental, Facultad de Farmacia, Universidad de Concepción, Concepción 4030000, Chile; lbustamante@udec.cl

**Keywords:** pentacyclic triterpenes, ceanothic acid, acetylcholinesterase

## Abstract

Alzheimer’s disease (AD) is a multifactorial and fatal neurodegenerative disorder. Acetylcholinesterase (AChE) plays a key role in the regulation of the cholinergic system and particularly in the formation of amyloid plaques; therefore, the inhibition of AChE has become one of the most promising strategies for the treatment of AD, particularly concerning AChE inhibitors that interact with the peripheral anionic site (PAS). Ceanothic acid isolated from the Chilean Rhamnaceae plants is an inhibitor of AChE through its interaction with PAS. In this study, six ceanothic acid derivatives were prepared, and all showed inhibitory activity against AChE. The structural modifications were performed starting from ceanothic acid by application of simple synthetic routes: esterification, reduction, and oxidation. AChE activity was determined by the Ellmann method for all compounds. Kinetic studies indicated that its inhibition was competitive and reversible. According to the molecular coupling and displacement studies of the propidium iodide test, the inhibitory effect of compounds would be produced by interaction with the PAS of AChE. In silico predictions of physicochemical properties, pharmacokinetics, drug-likeness, and medicinal chemistry friendliness of the ceanothane derivatives were performed using the Swiss ADME tool.

## 1. Introduction

The most common neurodegenerative diseases (NDs) include Alzheimer’s disease (ND), Parkinson’s disease, prion disease, amyotrophic lateral sclerosis, motor neuron disease, Huntington’s disease, spinal muscular atrophy, and spinocerebellar ataxia [1,2]. AD is a neurodegenerative disease that is responsible for approximately 70% of cases of dementia and is characterized by loss of memory and other cognitive disabilities [3,4,5,6]. Despite the different and varied investigations, the exact molecular mechanism involved in AD initiation and progression is still unclear. Thus far, the available data clearly suggest that AD is a complex, multifactorial disorder Please note that the size of figures will be adjusted appropriately to ensure the clarity and readability of the image. Changes to the position of figures and tables may occur during the production stage, and advanced age is the most significant risk factor. The neurodegenerative process could be due to three causes: the accumulation of amyloid beta peptide deposit (AB); alteration and accumulation of hyperphosphorylated protein tau; and oxidative stress in the brain. These factors lead to synaptic dysfunction and neurodegeneration [7,8,9]. In Alzheimer’s disease, the sum of several biochemical and cellular events that are not yet fully understood invariably leads to the loss of cholinergic neurons in the basal forebrain nuclei [10]. These processes produce a progressive reduction of the neurotransmitter acetylcholine (ACh), which produces loss of memory and cognitive deficit [11,12].

Acetylcholinesterase (AChE) accelerates the evolution of AD by two possible mechanisms. First, AChE is responsible for the hydrolysis of ACh, which occurs in the catalytic active site (CAS). According to the cholinergic hypothesis, an average 55% decrease in cholinergic signaling is observed in the brain of patients with AD compared with normal individuals. Moreover, cholinergic neurotransmission is involved in learning and memory consolidation [13]. These facts suggest that cholinergic modulation would be a beneficial strategy for treating AD [14]. The results obtained in double-transgenic mice by Rees and coworkers support the notion of a cholinergic–amyloid interrelationship with the symptomatology of AD, particularly with memory loss [15]. On the other hand, the peripheral anionic site (PAS) generates a stable complex with β-amyloid peptide (Aβ) and by this means expedites the oligomerization of Aβ peptides and aggregation of senile plaques. Bartolini and co-workers demonstrated that interaction between HuAChE and β-amyloid produced the formation of Aβ fibrils. Besides the aggregation of β-amyloid perhaps being inhibited by propidium, added to the above different experiments, research has also shown that the interaction between AChE and Aβ results in aggregation of Aβ. Interestingly, this interaction cannot be reversed by edrophonium—a CAS inhibitor—but can be reversed by propidium iodide, a PAS inhibitor [16,17,18,19,20,21,22,23].

In actuality, only four approved drugs are available that can help improve symptoms, including three acetylcholinesterase (AChE) inhibitors (donepezil, rivastigmine, and galantamine) and one N-methyl-D-aspartate (NMDA) antagonist (memantine) [3]. These drugs do not have curative effects; they only decrease or attenuate the symptomatology. Therefore, it has become necessary to develop drugs that may delay the onset, slow the progress, or treat the symptoms of AD.

**Figure 1:**
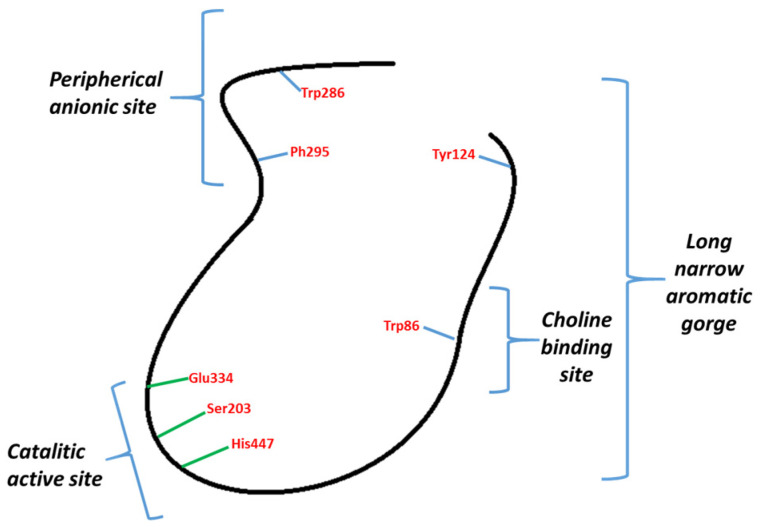
General representation of AChE.

Inhibitors of AChE may be classified into two types: those binding to CAS and those binding PAS (Figure 1). Galantamine binds to CAS and donepezil to CAS and PAS. These have attracted attention, given that it has been described how AChE through PAS interacts with Aβ peptides, stimulating their aggregation [24,25]. The reduction of its formation, build-up, and aggregation are some of the main goals in the development of a cure for AD.

In previous studies carried out in plants of the Rhamnaceae family that grow in Chile, we demonstrated that these plants have significant insecticidal activity, and this activity is due to the presence of compounds with acetylcholinesterase inhibitory activity [26]. The Lineweaver–Burk plots revealed that natural ceanothanes isolated from the Rhamnaceae plants family are typically mixed AChE inhibitors similar to that of donepezil [27]. Keeping in mind the relationship between PAS and the formation of senile plaques [28] and the presence in the Rhamnaceae family of plants with metabolites showing the ability inhibit AChE by interaction with PAS, we considered it interesting to study the effect of the chemical modification of ceanothic acid on the inhibitory activity of this compound on AChE and its interaction with PAS because molecules that act in this way offer a good potential for better treatment of AD.

## 2. Results

Plants of the Rhamnaceae family, particularly those that grow in Chile, are characterized by the presence of two types of secondary metabolites: triterpenes and alkaloids. In particular, the triterpenes have ceanothane and dammarane skeletons. These plants possess antibacterial, cytotoxic, antiprotozoal, and insecticidal activity. Ceanothic acid is the main component, and it is possible to isolate it in sufficient amounts to develop structural modification with the purpose of evaluating its biological activity, particularly the capacity to inhibit the acetylcholinesterase enzyme. The structure of all compounds was determined by nuclear magnetic resonance (^1^H and ^13^C NMR) and mass spectrometry.

### 2.1. Synthesis of Ceanothic Acid Derivatives

**Figure 2:**
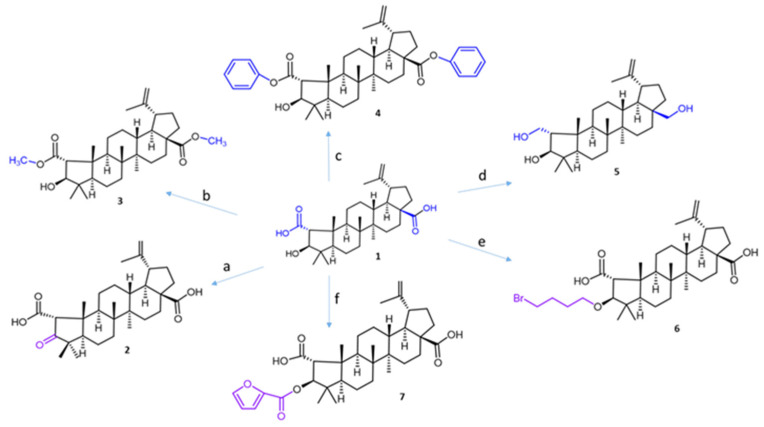
The synthesis routes for ceanothic acid derivatives. Reagents and conditions: (**a**) Jones reagent, acetone, 0 °C; (**b**) CH_3_I, K_2_CO_3_, acetone, rt; (**c**) K_2_CO_3_, DMF, benzyl bromide, 12 h, rt; (**d**) LiAlH_4_, THF, rt; (**e**) 1,4-dibromobutane, K_2_CO_3_, DMF, 60–70 °C; (**f**) furoyl chloride, DMAP, 70°. The number corresponds to the derivative compounds from ceanothic acid. The color stands out the modification made.

Ceanothic acid **1** was isolated from the roots of *Colletia hixtrix* (Rhamnaceae) and was used as a precursor to obtain different derivatives (Figure 2). The IR spectrum of ceanothic acid shows absorption at 2500–3100 cm^−1^ and 1698 cm^−1^ for the carboxylic acid function. ^1^H NMR showed C-2 H 3.18 (s) and C-3H, 4.99 (bs). Ceanothic acid reveals two carboxyls: one at 178.7 and another at 177.9, assignable to C-28 and C-1, respectively. The signals at 151.3 and 110.8 are assigned to C-20 and 29 vinyl carbons. The signal at 65.69 is due to C-2, and the signal at 85.0 is due to C-3.

Compound **2** was obtained by oxidation of ceanothic acid with Jones reagent at 0 °C. The IR spectrum shows absorption at 3400–3250 (COOH), 2915, 2849, 1708 (C=O), 1588, 1381, and 1026 cm^−1^. ^1^H NMR is very similar to ceanothic acid **3**, showing the disappearance of the signal 4.99 ppm; it corresponds to the H-3 product of the oxidation of the hydroxyl group in the same position. This correlates with the appearance of a signal in the ^13^C NMR at 216.6 ppm corresponding to C=O in C-3.

Compound **3** was obtained by methylation of the carboxylic groups at C-1 and C-28 of ceanothic acid with CH_3_I. ^1^H-NMR shows a singlet at 3.65 ppm as a characteristic signal, which integrates to six protons, which is why it is assignable to the new two methyl groups of the ester that is formed, which confirmed the structure of the derivative. The mass spectrum of compound 3 shows an [M + H]^+^ fragment at 515.38 *m*/*z* consistent with the introduction of two methyl groups.

Compound **4** was obtained by esterification of the carboxylic groups at C-1 and C-28 of ceanothic acid with benzyl bromide. The pseudomolecular ion [M + H]^+^ of the compound is observed at *m*/*z* 639.4539, which agrees with the theoretical molecular mass of 638.40. The ^13^C-NMR spectrum shows two signals at 175.80 ppm and 174.75 ppm corresponding to carbons C-1 and C-28. The aromatic carbon signals at 136.47 ppm and 135.5 ppm plus a set of signals between 128.7 ppm and 128.09 ppm, are attributable to the benzene ring. Other important signals are those corresponding to –CH_2_- of benzyl, which appear at 66.76 ppm. The ^1^H-NMR of this compound presents a multiplet between 7.30 ppm and 7.36 ppm corresponding to the protons of the aromatic ring of the benzyl groups. The double doublet between 5.15 ppm and 5.05 ppm corresponds to the protons of –CH_2_- of benzyl.

Compound **5** was obtained by reduction of carboxylic groups at C-1 and C-28 by treatment of ceanothic acid **1** with AlLiH_4_ in diethylether. The ^13^C NMR spectrum shows the disappear signals corresponding to carboxylic groups at 177 ppm and 178 ppm. Also, the ^13^C NMR spectrum of compound **5** shows three signals corresponding to carbon binding to oxygen at C-1, C-3, and C-28 (59.36, 56.30, and 55.53 ppm, respectively). 

Compound **6** was obtained by reaction of ceanothic acid with 1,4-dibromobutane in the presence of K_2_CO_3_ in DMF to give the corresponding C-3 ether in 65–80% yield.

Compound **7** was obtained by reaction between ceanothic acid with 2-furoyl chloride. The ^13^C NMR shows a signal at 158.13 ppm assignable to the carboxylic group of furanoyl. Additionally, four new signals to 146.53 ppm, 144.60 ppm, 117.94 ppm, and 111.83 ppm can be observed. These signals are assigned to methine carbons and quaternary carbon of the furanoate. ^1^H NMR shows the characteristic signals of the ceanothane skeleton, to which are added aromatic signals to 7.0 ppm, 7.52 ppm, and 7.69 ppm corresponding to CH of the furanoate group.

### 2.2. Enzyme Inhibition and Kinetics Assays

**Figure 3:**
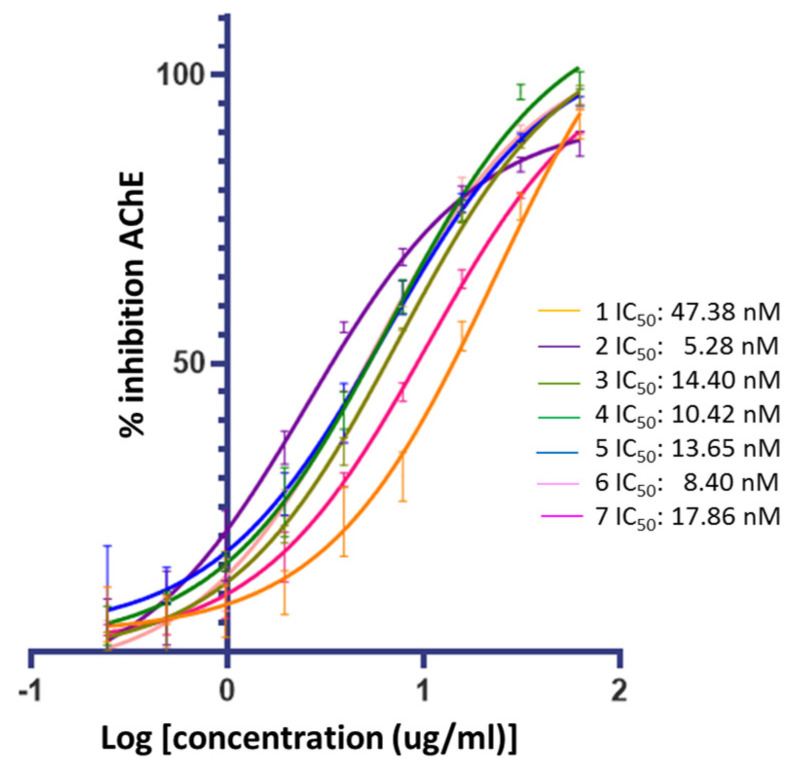
Inhibitory activities of ceanothane compounds **1**–**7** on AChE from *Electroporus electricus*.

Figure 3 confirms that ceanothic acid derivatives retain AChE inhibitory activity like their precursor. On the other hand, the compounds derived from ceanothic acid present an inhibitory activity higher than the precursor, showing IC_50_ between 5.28–17.86 nM (Table 1). These results were compared with the IC_50_ values of ceanothic acid (47.38 nM), the classic competitive inhibitor galantamine (IC_50_ = 101 nM), and the AchE mixed inhibitor donepezil (IC_50_ = 12 nM). The IC_50_ was calculated as the average of the results obtained in triplicate in the assay.

**Figure 4:**
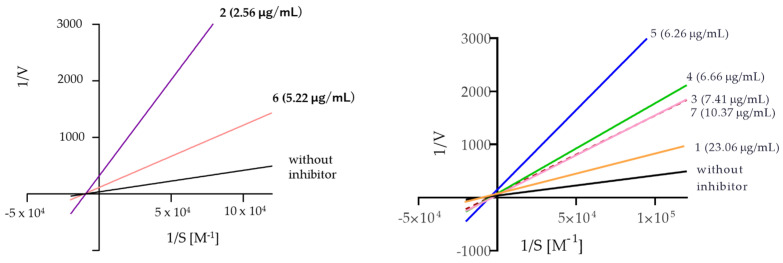
The Lineweaver–Burk reciprocal plots of the initial velocity in the function of substrate concentrations.

The analysis of the Lineweaver–Burk plots (Figure 4) for the compounds assayed showed that compounds **3**, **4**, **5**, and **7** are mixed inhibitors, while compounds **2** and **6** are noncompetitive inhibitors. The above was inferred from the calculation of alpha, whose value is greater than one, meaning that these compounds (**3**–**5** and **7**) can act with greater affinity on the free enzyme compared to the enzyme–substrate complex. Moreover, compound **7** has a very large alpha value, suggesting an exclusive preference for the free enzyme; therefore, its binding mode is much closer to that of a competitive inhibitor compared to the **3**–**5** inhibitors. 

### 2.3. Displacement of Propidium Iodide from the PAS of EeAChE

The AChE enzyme has two sites (CAS and PAS) that interact with the inhibitors of this enzyme [24,25,28,29]. By interacting with soluble β-amyloid peptides and facilitating their aggregation, the AChE peripheral anionic site (PAS) at the gorge’s edge contributes to AChE’s pro-aggregation capacity toward β-amyloid. Propidium iodide (PI) is a selective ligand for the PAS, showing a significant decrease in AChE-induced Aβ aggregation [15]. The potential ability of compounds to bind PAS and thus block the pro-aggregation activity of AChE is possible to evaluate through competitive propidium displacement from the PAS of AChE. Propidium iodide exhibits a fluorescence increase upon binding to AChE. A decrease in PI fluorescence in the presence of test compounds indicates that they are able to displace propidium and bind to the PAS of AChE, suggesting that they would thereby block the AChE-mediated aggregation of β-amyloid [28,30]. Donepezil is a mixed-type AChE inhibitor, which has the demonstrated ability to block AChE-PAS-induced Aβ aggregation, which is why it was used as the reference compound for this assay [15]. The results are shown in Table 2, where it can be seen that all the ceanothic acid derivatives produced a displacement of PI from the AChE PAS in a range of 24 to 40%. The most prominent effect on the displacement of PI from the PAS of AChE was found for compound **5**. Taken together, the results from propidium displacement, kinetics, and molecular docking (see Section 2.4) indicate that the ceanothic acid derivatives are dual-site AChE inhibitors occupying both CAS and PAS pockets. Thanks to their binding to the PAS of AChE, they can potentially block AChE-induced aggregation of β-amyloid, thus having an additional positive disease-modifying effect.

### 2.4. Docking Studies

The docking binding energy for the compounds (**1**–**7**) had a good correlation with the experimental IC_50_ values (0.8831 Pearson products at the 95% confidence level) (Table 1). In general, all the derivatives prepared, in accordance with the results obtained in the enzymatic tests, have a better binding energy than ceanothic acid with PAS. Furthermore, like their precursor, the derivatives maintained the same π-sigma, π-alkyl, and alkyl-type interactions with the PAS amino acids (Tyr72, Tyr124, Trp286, Trp341, and Phe338). Molecular docking showed that ceanothic acid binds via hydrogen bonds with residues close to the aromatic cavity, such as Ser293, Phe295, Arg296, and Tyr337, and has π-sigma π-alkyl, and alkyl-type interactions with other PAS residues [27]. The binding energy was −7.46 kcal/mol. 

**Figure 5:**
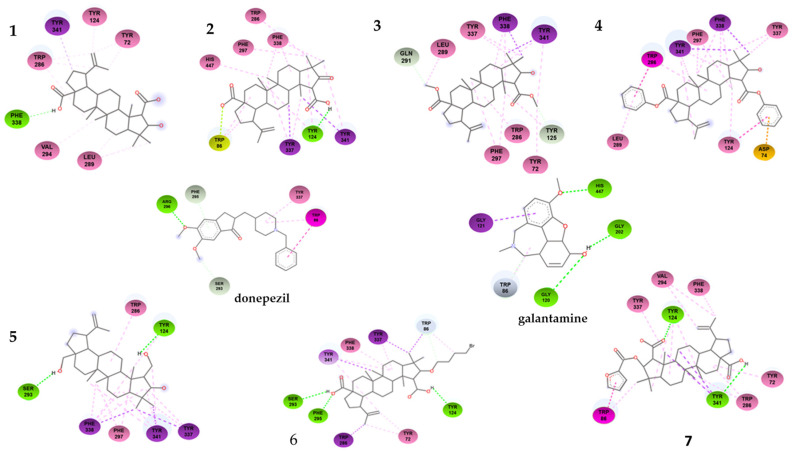
Two-dimensional binding interactions of compound **1**–**7** with the best docking energy values. Hydrogen bonds are highlighted in green and hydrophobic interactions in pink.

As shown in Table 1, the compounds that showed the best inhibitory activity of the acetylcholinesterase enzyme were compounds **2** and **6**. The enzyme kinetics assays showed that both compounds would act as non-competitive inhibitors of the enzyme. This information is supported by the molecular docking study since the binding sites of these compounds to the enzyme correspond to amino acids located in the PAS. As can be seen in Figure 5 and in the Table 3, compound **2** forms a hydrogen bond with residue Tyr124, in addition to a π-lone pair interaction with residue Trp86 and several π-alkyl and alkyl-type interactions. The compound showing the highest negative downlink energy was compound **6**, as it was linked to the enzyme through conventional hydrogen bonds with residues Tyr124, Phe295, and Ser293 and one carbon–hydrogen bond with the Trp 86 residue in addition to having several pi-type and alkyl interactions. 

Although derivatives **3** and **4** lose hydrogen bonding after the loss of the -OH group of the carboxyls, other interactions arise that do not impair their binding to PAS. Compound **3** shows a carbon–hydrogen bond with residues Gln291 and Tyr125 and several hydrophobic π-sigma and π-alkyl interactions. On the contrary, compound **4** did not show hydrogen-bond-type interactions. The reduction of -COOH to -CH_2_OH in derivative **5** significantly increases its binding energy (−10.08 kcal·mol^−1^), allowing more hydrogen-bond-type interactions. Derivative **7** did not improve its binding energy (−7.34 kcal·mol^−1^) with respect to ceanothic acid (−7.46 kcal·mol^−1^). Unlike the other derivatives, it presents a stacked π–π-type bond (interaction between the pi groups of the aromatic rings) with Trp86, a residue belonging to the anionic subsite of the CAS. These results are related to a certain extent with the results of the enzymatic assays, with derivative **7** being the only mixed-competitive-type inhibitor.

Finally, derivative **6** (−10.81 kcal·mol^−1^) does not present major changes in the interactions with the amino acids of PAS compared to other derivatives. However, the bromobutyl substituent added at C-3 interacts with Trp84, which indicates that this derivative, while interacting with PAS, arranges itself in such a way that the bromobutyl substituent is located towards the interior of the enzyme, being able to interact with this amino acid of the anionic subsite.

Docking focused on the CAS, specifically the catalytic triad, as expected, did not give positive results. The binding energies of the derivatives gave very high values, an indicator that the interactions do not occur spontaneously, and an excess of energy would be required for this to happen. These results support the idea that these compounds would act as specific PAS inhibitors.

### 2.5. In Silico ADME Predictions for Ceanothanes Derivatives

In silico predictions of physicochemical properties, pharmacokinetics, drug-likeness, and medicinal chemistry friendliness of ceanothanes derivatives were performed using the free access Swiss ADMET boost, SwissADME, and ADMET prediction service [2,31,32]. The information obtained in this way gave the first evaluation of ceanothanes derivatives that could be considered drugs in AD treatment. As can be seen (Table 4), all ceanothanes derivatives show high predicted values for intestinal absorption, enabling their oral administration. In the same way, the compounds show values of logBB that indicate an optimal-to-moderate capacity to cross the blood–brain barrier (BBB). The cardiac toxicity risk (pKi and pIC_50_) for all analyzed compounds (2.4–6.5 log units) are in the lower or medium part of their possible range (3–9 log units). The estimated drug-likeness (ED) of assayed compounds is a measure of the possible degree of interaction; our results show a null level of interaction. Lipophilicity, as evaluated by the octanol/buffer solution distribution coefficient at pH = 7.4 (logD7.4), is a major determinant of various absorption, distribution, metabolism, elimination, and toxicology (ADMET) parameters of a drug candidate, and the compounds assayed present values considered optimal. According to the values shown by the ceanothanes derivatives for hERG, these compounds would present cardiotoxicity.

## 3. Discussion

Alzheimer’s disease is a complex, multifactorial disease. Though the etiology of AD remains to be elucidated, multifaceted pathological features are believed to occur in a sequential but overlapping manner [31]. Thus, the development of multifunctional agents that could modify different pathologies simultaneously would be an ideal strategy to tackle this disease [32]. Dual inhibitors of Aβ aggregation and cholinesterase are emerging as promising multi-target ligands to modify the course of AD [2,33,34]. Previously, it was reported that natural ceanothanes isolated from different plants of the Rhamnaceae family possess an interesting inhibitory activity on AChE, and this activity occurs by interaction with CAS and PAS sites in the enzyme [26].

Despite continuing debate about the amyloid β-protein (or Aβ) hypothesis, new lines of evidence from laboratories and clinics worldwide support the concept that an imbalance between the production and clearance of Aβ42 and related Aβ peptides is a very early, often initiating factor in Alzheimer’s disease (AD). Although many factors contribute to AD pathogenesis, Aβ dyshomeostasis has emerged as the most extensively validated and compelling therapeutic target [35]. Histochemical studies have demonstrated that the AChE associated with senile plaques differs enzymatically from the enzyme associated with normal nerve fibrils and neurons in several respects. Different authors have shown that AChE promotes the assembly of Aβ into amyloid fibrils and that a monoclonal antibody directed against the PAS of AChE inhibits the effect of the enzyme upon amyloid formation [36,37,38]

AChE has been described in cholinergic and non-cholinergic processes in both the central and peripheral nervous systems [39,40]. The enzyme is secreted and becomes associated with extracellular structures, namely the synaptic basal lamina at the neuromuscular junction and the amyloid plaques of AD brain. Several anticholinesterase drugs can decrease the effect of AChE on amyloid formation. Experimentally, it has been found that using inhibitors of AChE such as tacrine, galantamine, or edrophonium, whose mechanism of action is by interaction with CAS, gives no observed effect on Aβ plate formation. On the contrary, when they are used anticholinesterase agents whose mechanism of action is by interaction with the PAS of enzymes such as propidium, fasciculin, or donepezil, Aβ formation is blocked. These findings are entirely consistent with studies carried out using a monoclonal antibody directed against the PAS site of AChE [41].

Our results show the structural modification of ceanothic acid yields molecules with interesting inhibitory activity against AChE. Kinetic, fluorescence, and docking data show that the inhibitors studied interact with PAS and CAS, but the energy of the interaction with the latter is very high, which indicates that the residence time in CAS is very short, the interaction with PAS is therefore more effective. This type of interaction with AChE is similar to donepezil: The compounds have a dual action, increasing the level of ACh and decreasing Aβ peptide formation in the neuron. 

Lipinski’s rule states that, in general, an orally active drug can have no more than one violation of the criteria established by this rule. The compounds assayed show two breaches of the rule: molecular weight and liposolubility (MLOGP > 4.15). As shown in Table 1, the molecular weight of the compounds is between 450–640 daltons, with 500 daltons being the maximum value established. Therefore, with the results of ADME prediction and SwissADME, is possible to think that these molecules are good models for the development of CNS oral drugs [42,43,44,45].

Thus, the development of multifunctional agents that could modify different pathologies simultaneously would be an ideal strategy to tackle this disease [2]. It has been suggested that dual inhibitors of Aβ aggregation and cholinesterase are emerging as promising multi-target ligands to modify the course of AD [35,46].

## 4. Materials and Methods

### 4.1. Chemicals

NMR spectra were recorded on a Bruker spectrometer 400 MHz (Palo Alto, CA, USA) using CDCl_3_ as the solvent and TMS as the internal standard. Chemical shifts are reported in units (ppm) and coupling constants (J) in Hz. IR spectra were recorded on a Shimadzu FTIR-8400 infrared spectrophotometer. Silica gel (Kieselgel-mesh 0.15/0.30, Merck, Darmstadt, Germany) was used for all liquid chromatography procedures (LC). For thin-layer chromatography (TLC), silica gel GF254 was used as the stationary phase with a plate dimension of 20 cm × 20 cm × 0.20 mm for analytical TLC (Merck, Darmstadt, Germany) and 20 cm × 20 cm × 0.25 mm for semi-preparative TLC (SPTLC) (Merck, Darmstadt, Germany). Spots on the chromatogram were visualized under UV light and by spraying with 5% H_2_SO_4_ in methanol and then heating at 110 °C for 5 min. Acetylcholinesterase (from *Electrophorus electricus*), 5,50-dithiobis-(2-nitrobenzoic acid) (DTNB), and acetylthiocholine iodide were purchased from Sigma-Aldrich (St. Louis, MO, USA).

### 4.2. Synthetic Methodology

#### 4.2.1. Preparation of 3-oxo-Ceanothic Acid (**2**)

Ceanothic acid 3 (0.05 g, 0.1 mmol) was previously dissolved in CH_2_Cl_2_ (10 mL) and then was added to a 0.04 M pyridinium chlorochromate in CH_2_Cl_2_ solution (40 mL). The resulting solution was stirred at room temperature for 2 h, and then, Et_2_O (30 mL) was added. The resulting brown solid residue was removed by filtration through a Celite pad, and evaporation of the filtrate yielded a residue that was purified on a silica gel column (120 g, 230–400 mesh) eluted with n-hexane:EtOAc (80:20) to give **6** (0.036 g, yield 72%).

#### 4.2.2. Preparation of Ceanothic 2, 28-Dimethyl Ester (**3**)

A mixture of ceanothic acid (5.1 mg), K_2_CO_3_ (80 mg), CH_3_I (300 μL), and acetone (1 mL) was stirred for 72 h at room temperature. The reaction mixture was poured over distilled water (14 mL), and the resulting suspension was extracted twice with EtOAc (4:1, *v*/*v*). The organic layer was dried over anhydrous Na_2_SO_4_ and evaporated to produce 4.6 mg of the crude esterified product, which was purified by column chromatography (hexane/Me_2_CO 9:1) to give **3** (4.2 mg, 91.3% yield) as a white powder.

#### 4.2.3. Preparation of Compound 1,28-Dibenzyl-ceanothate (**4**)

Ceanothic acid (70 mg, 0.14 mmol) was dissolved in dimethylformamide (DMF) (10 mL), and then, benzyl bromide (72 mg, 0.42 mmol) and K_2_CO_3_ (275 mg, 0.2 mmol) were added, and the mixture was stirred for 4 h at 85 °C. This reaction was monitored by TLC. The reaction solution was washed with water, filtered, and evaporated with vacuum.

#### 4.2.4. Preparation of 2,3,28-Trihydroxy-ceanoth-20(30)-ene (**5**)

Anhydrous THF (4 mL) was injected to LiAlH_4_ (40 mg, 0.53 mmol) under nitrogen. The suspension was stirred at room temperature for 10 min, and then, compound 1 (40 mg, 0.06 mmol), dissolved in anhydrous THF (1 mL), was added dropwise. The mixture was heated under reflux for 2.5 h. After cooling, hydrated Na_2_SO_4_ was added to the suspension to destroy excess LiAlH_4_. The suspension was then filtered through a Celite pad, and the filtered residue washed with EtOAc. The organic phase was evaporated to give a residue that was purified by preparative HPLC with a yield of 89%.

#### 4.2.5. Synthesis of 3-(4-Bromobutoxy)-ceanothic Acid (**6**)

Ceanothic acid (**1**) (0.1 g, 0.2 mmol) was dissolved in dimethylformamide (DMF, 15 mL), and anhydrous potassium carbonate (0.027 g, 0.2 mmol) was added to the solution. The reaction mixture was refluxed at 70 °C with 1,4-dibromobutane (0.04318 g, 0.2 mmol) with continuous stirring for 3 h. After the completion of the reaction (monitored by TLC), the mixture was filtered, diluted with water, and extracted with ethyl acetate (EtOAc). Ethyl acetate solution was concentrated and purified through by column chromatography, eluting with pure chloroform to give compound **6** with a 60% yield.

#### 4.2.6. Synthesis of 3-O-Furoyl-ceanothic Acid (**7**)

Ceanothic acid (40 mg, mmol) was dissolved in anhydrous-pyridine (0.9 mL). Then, 30 µL of furoyl chloride and 30 mg of DMAP were added. The mixture was stirred at 70 °C for 2 h and then stored at room temperature overnight. Afterwards, the reaction mix was diluted with EtOAc (1 mL), and the organic phase was washed with aqueous solution of HCl (0.1 M). The organic phase was dried with anhydrous MgSO_4_ and concentrated at reduced pressure. The compound **7** was purified by preparative HPLC (yield 85%).

### 4.3. In Vitro AChE Inhibitory Activity Assay

The Ellman assay was used to test acetylcholinesterase (AChE from *Electrophorus electricus*) inhibition activity [47]. A mixture of the DTNB (125 µL), enzyme solution (25 µL), and compound solution (25 µL) was prepared and incubated at room temperature for 20 min. All the assays were under 0.1 M KH_2_PO_4_/K_2_HPO_4_ buffer, pH 8.0. The substrate was added to start the enzymatic reaction. The absorbance (λ = 405 nm) was recorded at a controlled temperature of 30 °C for 5 min. All measurements were performed fivefold as triplicate. The compounds were assayed in a dilution interval of 15 to 500 µg/mL. Galantamine was used as positive control. The percentage of inhibition was determined as follows:

$$I\left(\%\right)=\left(1-\frac{A_{probe}}{A_{blank}}\right)100$$

### 4.4. Kinetic Characterization of AChE Inhibition

To investigate the inhibition mechanism of the tested compounds on AChE, a kinetic analysis was performed. The experiments were carried out using a combination of four substrate concentrations and three inhibitor concentrations with the aim to obtain a double reciprocal plot (Lineweaver–Burk), in which each point is the mean of three different experiments. A parallel control with no inhibitor in the mixture allowed adjusted activities to be measured at various times.

### 4.5. Propidium Iodide Displacement Assay

This trial was carried out to evaluate the interaction of the ceanothanes triterpenes under study with the PAS of AChE. A solution of the test compound or standard donepezil was incubated with five units of EeAChE at 25 °C for 15 min. After incubation, 50 µL of 1 µM propidium iodide solution was added to yield the final assay volume of 200 µL. After 15 min, the fluorescence intensity was observed at an excitation wavelength (λex = 535 nm) and an emission wavelength (λem = 595 nm) using a fluorescence plate reader Perkin Elmer VictorX2 (Perkin Elmer, Singapore). The percentage inhibition was calculated by the following expression: 100 − (IFi/IF_0_ × 100), where IF_1_ and IF_0_ correspond to fluorescence intensities with and without the test compound, respectively. Each assay was carried out in triplicate independent experiments.

### 4.6. In Silico Assays

#### 4.6.1. Construction of the Ligands

To construct the three-dimensional models of the ligands, the Avogadro 1.2.0n program was used. The geometry of the ligands was optimized, and their energy was minimized using the MMFF94 force field, ideal for organic compounds molecular docking.

#### 4.6.2. Molecular Docking

To carry out the molecular docking, the X-ray diffraction crystallized structure of human AChE (PDB ID: 6O4X; Resolution: 2.29 Å [48]) obtained from Protein Data Bank was used. To select the possible anchoring sites of the ligands to the protein, an alignment was carried out with AChE from *Torpedo californica* (PDB ID: 1EVE; Resolution: 2.50 Å [49]) through VMD 1.9.4 software; in this way, the amino acids belonging to the active sites of the enzyme described previously were selected. The crystal structure of the enzyme was edited in the Discovery Studio Visualizer software (v17.2.0.16349 2016), in which the ligands and water molecules associated with the PDB file were removed.

To perform the molecular docking, the AutoDock 4 software was used, using the Lamarckian genetic algorithm [50] and assuming rigid ligands in the macromolecule and total flexibility for inhibitors. This program evaluates the binding affinity of the protein and the ligand to choose the best complex where the binding energy is the lowest. The method was revalidated using galantamine and donepezil as controls. Kollman charges were added to the enzyme, and Gasteiger charges were added to the ligands in addition to the polar hydrogens. Docking was carried out in two areas in the rigid enzyme: the PAS and the CAS. The grid map for PAS was located at the entrance of the enzyme throat considering the main amino acids belonging to PAS (*T. californica*: Tyr70, Asp72, Tyr334, Trp279, and Phe331; human AChE: Tyr72, Asp74, Tyr341, Trp286, and Phe338). For evaluation of the interaction of the compounds with AChE, docking focused on the amino acids of the catalytic triad (*T. californica*: Ser200, Glu327, and His440; human AChE: Ser203, Glu334, and His447) and the amino acid of the PAS (*T. californica*: Phe330, Trp84, and Tyr121; human AChE: Tyr337, Trp86, and Tyr124). Searching parameters were to 50 runs and with a maximum number of 25.000.000 evaluations for each ligand. The RMSD threshold for multiple clustering was set to ˂0.5 Å. 

The results were ordered according to the binding energy and possible conformations. The lowest binding energy and the most probable conformation were chosen for future analyses. Discovery Studio Visualizer software was used to acquire a two-dimensional image of the chosen conformation.

#### 4.6.3. In Silico Prediction of ADME

In silico predictions of ADME properties for ceanothanes derivatives were performed using the free access Swiss-ADME tool (http://www.swissadme.ch/, accessed on 5 February 2024) [51,52].

## 5. Conclusions

In summary, our study revealed that ceanothane derivatives present better inhibitory activities on the enzyme AChE than ceanothic acid used as a precursor. Docking and displacement studies show that inhibitory activity occurs preferably by PAS interaction, given that the energy interaction with CAS is very high. These types of compounds are promising candidates for further development as potential drugs in the treatment of Alzheimer’s disease. Computational ADMET profiles predicted that all the conjugates should have good intestinal absorption, medium blood–brain barrier permeability, and medium cardiac toxicity risks. Moreover, the PAINS filter check for the compounds did not identify any alerts.

## Figures and Tables

**Table 1:** Inhibition of acetylcholinesterase (AChE from *Electrophorus electricus*) from Ellman’s assays, IC_50_, Km, Vmax, and binding energy for ceanothanes **1**–**7** used in this study.

	M (g/mol)	IC_50_ ± SEM		Km (nM)	Vmax	α	Binding Energy (kcal·mol^−1^)
Compounds		µg/mL	nM				
**1**	486.69	23.06 ± 1.63	47 ± 3				−7.46
**2**	484.68	2.56 ± 0.40	5.3 ± 0.8	0.1	0.0032	1.01	−8.66
**3**	514.75	7.41 ± 0.26	14 ± 0.5	0.2	0.0133	2.98	−10.03
**4**	638.89	6.66 ± 0.72	10 ± 1.1	0.1	0.0131	3.49	−10.29
**5**	458.73	6.26 ± 0.74	14 ± 1.6	0.2	0.0066	2.35	−10.08
**6**	621.70	5.22 ± 0.56	8 ± 0.9	0.1	0.0086	0.98	−10.81
**7**	580.76	10.37 ± 0.33	18 ± 0.6	0.3	0.0205	10.39	−7.34
**Galantamine**	287.35	0.29 ± 0.02	101 ± 0.7				

**Table 2:** Displacement of propidium iodide by ceanothic acid derivatives **1**–**7**.

Compounds	% Displacement of Propidium Iodide (Mean ± SEM)
**1**	24.24 ± 0.32
**2**	28.71 ± 0.99
**3**	27.01 ± 0.59
**4**	30.56 ± 0.83
**5**	39.03 ± 0.38
**6**	31.51 ± 1.38
**7**	28.50 ± 2.55
**Donepezil**	82.50 ± 0.36

**Table 3:** Interactions between ligands and human acetylcholinesterase.

Compound	Interactions	*h*AChE Residues
**1**	Hydrogen bond	Phe338
	π-sigma	Tyr341
	π-alkyl	Tyr134; Tyr72; Trp286; Val294; Leu289
**2**	Hydrogen bond	Tyr124
	π-sigma	Tyr337; Tyr341
	π-alkyl	Phe338; Trp286; Phe297; His447
	π-lone pair	Trp86
**3**	Carbon–hydrogen bond	Gln291; Tyr125
	π-sigma	Phe338; Tyr341
	π-alkyl	Tyr337; Leu289; Trp286; Phe297; Tyr72
**4**	π-anion	Asp74
	π-sigma	Phe338; Tyr341
	π-π T-shaped	Trp 286; Tyr124
	π-alkyl	Leu289; Tyr124; Phe297; Tyr337
**5**	Hydrogen bond	Tyr124; Ser293
	π-sigma	Phe338; Tyr341; Tyr337
	π-alkyl	Phe297; Trp286
**6**	Hydrogen bond	Tyr124; Phe295; Ser293
	Carbon–hydrogen bond	Trp86
	π-π T-shaped	Tyr341; Trp286; Tyr337
	π-alkyl	Phe338; Tyr72
**7**	Hydrogen bond	Tyr124; Tyr341
	π-π T-shaped	Trp86
	π-alkyl	Phe338; Val294; Tyr337; Tyr72; Trp286
Galantamine	Hydrogen bond	His447; Gly202; Gly120
	Carbon–hydrogen bond	Trp86
	π-sigma	Gly121
Donepezil	Hydrogen bond	Arg296
	Carbon–hydrogen bond	Phe295; Ser293
	π-π T-shaped	Trp86
	π-alkyl	Trp86; Tyr337

**Table 4:** Predicted ADMET and physicochemical profiles of ceanothanes derivatives.

Compound	LogBB	HIA%	*h*ERG, pKi	*h*ERG, pIC_50_	LogPow	pS	logD_7.4_	QED	OB%	PGPI%
**1**	−1.74	67.75	3.52	2.8	6.01	−4.44	1.5	NO	35.84	31.77
**2**	−1.72	79.19	3.79	2.48	6.22	−4.33	1.52	NO	33.24	35.85
**3**	0.02	73.63	5.31	2.78	6.19	−4.24	1.76	NO	36.01	33.37
**4**	−1.27	100	6.5	3.01	9.05	−9.01	2.07	NO	32.13	39.45
**5**	−0.13	67.75	5.12	4.56	5.65	−4.26	1.41	NO	32.02	30.81
**6**	−1.43	79.19	3.5	3.09	8.21	−4.28	1.76	NO	32.28	32.21
**7**	−1.98	82.79	3.19	2.79	7.47	−4.35	1.93	NO	32.24	35.66

LogBB: blood–brain barrier permeability; HIA: human intestinal absorption (%); *h*ERG pKi: *h*ERG potassium channel affinity (−log[M]); *h*ERG, pIC50: *h*ERG potassium channel inhibitory activity (−log[M]); LogPow: octanol–water partition coefficient; pS: aqueous solubility (−log[M]); QED: quantitative estimate of drug-likeness; log D7.4: lipophilicity; OB: oral biodisponibility (%); PGPI%: P-glycoprotein inhibition (%).

## Data Availability

Data can be requested from the corresponding author due to restrictions on privacy.
